# Intermediate Monocytes but Not TIE2-Expressing Monocytes Are a Sensitive Diagnostic Indicator for Colorectal Cancer

**DOI:** 10.1371/journal.pone.0044450

**Published:** 2012-09-04

**Authors:** Dominic Schauer, Patrick Starlinger, Christian Reiter, Nikolaus Jahn, Philipp Zajc, Elisabeth Buchberger, Thomas Bachleitner-Hofmann, Michael Bergmann, Anton Stift, Thomas Gruenberger, Christine Brostjan

**Affiliations:** Department of Surgery, Medical University of Vienna, General Hospital, Vienna, Austria; UCLA, United States of America

## Abstract

We have conducted the first study to determine the diagnostic potential of the CD14++CD16+ intermediate monocytes as compared to the pro-angiogenic subset of CD14++CD16+TIE2+ TIE2-expressing monocytes (TEMs) in cancer. These monocyte populations were investigated by flow cytometry in healthy volunteers (N = 32) and in colorectal carcinoma patients with localized (N = 24) or metastatic (N = 37) disease. We further determined blood levels of cytokines associated with monocyte regulation. The results revealed the intermediate monocyte subset to be significantly elevated in colorectal cancer patients and to show the highest frequencies in localized disease. Multivariate regression analysis identified intermediate monocytes as a significant independent variable in cancer prediction. With a cut-off value at 0.37% (intermediate monocytes of total leukocytes) the diagnostic sensitivity and specificity ranged at 69% and 81%, respectively. In contrast, TEM levels were elevated in localized cancer but did not differ significantly between groups and none of the cytokines correlated with monocyte subpopulations. Of interest, *in vitro* analyses supported the observation that intermediate monocytes were more potently induced by primary as opposed to metastatic cancer cells which may relate to the immunosuppressive milieu established in the advanced stage of metastatic disease. In conclusion, intermediate monocytes as compared to TIE2-expressing monocytes are a more sensitive diagnostic indicator of colorectal cancer.

## Introduction

Monocytes are considered key players in innate immunity; they account for approximately 8–10% of human leukocytes and are characterized by the expression of the co-receptor CD14 for toll-like receptor 4 (TLR4) [1]. A small subset of human peripheral blood monocytes which co-expresses CD16 (Fcγ receptor III) has been identified in 1988 [2] and found to account for about 10% of total blood monocytes [3]. Heterogeneity within this CD16+ population was subsequently recognized [4]. The existence of 3 monocyte subpopulations based on the differential expression of CD14 and CD16 has recently been implemented into the new nomenclature of monocytes which distinguishes between the CD14++CD16- “classical”, the CD14++CD16+ “intermediate” and the CD14+CD16++ “non-classical” monocyte subset [5].

Recent studies on the gene expression profiles point to a developmental relationship between the three subsets with gradual changes in surface markers during maturation [6]–[7]. In comparison, *in vitro* culture and maturation of blood monocytes results in a gradual increase in CD16 expression [8]–[9] which is triggered by cytokines such as MCP-1 (monocyte chemoattractant protein 1), TGF-β (transforming growth factor beta), or M-CSF (macrophage colony stimulating factor) [10]–[12]. Furthermore, the three subpopulations exhibit distinct functional differences, with classical monocytes showing the highest phagocytosis potential [6]. In contrast, non-classical monocytes have a low capacity for phagocytosis, show a patrolling behavior along vessel walls and react strongly against nucleic acids and viruses [13]. The gene expression profile of intermediate monocytes has linked them to antigen processing and presentation, with inflammatory responses to bacterial pathogens and lipopolysaccharide (LPS) [6], [14]. Of interest, pro-angiogenic markers such as endoglin, vascular endothelial growth factor receptor 2 (VEGFR-2) and the angiopoietin receptor TIE2 are selectively overexpressed in the intermediate monocyte subset [6], [15].

TEMs (TIE2-expressing monocytes) have initially been described in mice to comprise a pro-angiogenic monocyte population that can enhance tumor growth by paracrine secretion of angiogenic factors such as VEGF and basic fibroblast growth factor [16]. Circulating TEMs are detected in the peripheral blood of healthy humans and cancer patients, and are predominantly found in the intermediate monocyte subset [15], [17]. They respond to angiopoietin-2 (ANG-2), a protein highly expressed in tumors, via the surface receptor TIE2 and its co-receptor TIE1 which can promote signaling by shedding a soluble TIE1 fragment [18]–[19]. Thus, TEMs preferentially accumulate at tumor sites including colorectal carcinoma but seem to be absent from normal tissue [17].

Monocyte subsets have been monitored in human blood in the context of diseases. However, the majority of studies did not discriminate between non-classical and intermediate monocytes but focused on the distinction between CD16 positive and negative subpopulations. Elevated levels of circulating CD16+ monocytes have been reported for pathological conditions such as sepsis [20], chronic hepatitis B [21], coronary artery disease [22], and cancer [23]. Hence, CD16+ monocytes have shown diagnostic and prognostic potential in inflammatory and malignant disease. More recent investigations illustrate that the further distinction between non-classical and intermediate monocytes may offer improved information. In particular, intermediate monocytes as opposed to non-classical monocytes were shown to be selectively elevated in severe asthmatic patients [24] and to predict adverse outcome in patients at high cardiovascular risk [25]. Comparable analyses in cancer patients are missing to date.

Given the novel insight into the distinct properties of intermediate monocytes and the pro-angiogenic (pro-tumor) phenotype of TEMs we hypothesized that these monocyte populations may possess diagnostic potential in colorectal cancer (CRC). We expected TEMs and intermediate monocytes to be significantly elevated in the peripheral blood of patients with CRC and to be further increased in advanced disease. To address this subject we conducted an explorative study on 93 participants including healthy volunteers, CRC stage I-III patients (localized colorectal cancer) and CRC stage IV patients (metastasized colorectal cancer, mCRC). We characterized the subpopulations of intermediate monocytes and TEMs in peripheral blood in relation to clinical parameters as well as to monocyte-associated cytokines including MCP-1, ANG-2, soluble TIE1 (sTIE1), and vascular endothelial growth factor A (VEGF-A). The intermediate monocyte subset as opposed to TEMs was found to be preferentially induced in early stages of disease and to constitute a novel, sensitive marker for colorectal cancer.

## Materials and Methods

### Ethics Statement

This non-interventional, clinical investigation was conducted according to the principles expressed in the Declaration of Helsinki. The analysis of blood samples was approved by the institutional “Ethics Committee of the Medical University of Vienna” (#331/2010 and #791/2010); all patients and healthy volunteers gave written informed consent.

### Patient Collective

Blood samples were collected from three subject groups: healthy volunteers (N = 32), patients with localized colorectal cancer (N = 24) and patients with metastasized colorectal cancer (N = 37). The majority of mCRC patients had their primary tumor resected and presented with liver metastases secondary to colorectal cancer. Blood was withdrawn before patient treatment, i.e. generally one day before surgery or immediately prior to neoadjuvant chemotherapy. The stage of disease according to the Union for International Cancer Control (UICC) was determined for all CRC patients following computed tomography scan and tumor resection. Healthy volunteers were apparently free of chronic and inflammatory diseases.

### Analysis of Monocyte Populations

Ethylene diamine tetra-acetic acid (EDTA) treated whole blood was processed at room temperature. Surface expression of CD14, CD16 and TIE2 was evaluated applying direct immunfluorescence staining followed by a lyse-no-wash procedure. In brief, 100 µl of whole blood were incubated with the following antibodies at saturating concentrations for 20 minutes: CD14-FITC (Becton-Dickinson, San Jose, CA, USA), CD16-PC5 (Beckman Coulter, Fullerton, CA, USA) and TIE2-PE (R&D Systems, Inc., Minneapolis, MN, USA). To eliminate erythrocytes, Versa Lyse solution (Beckman Coulter) was added for 20 minutes. Flow cytometry was immediately performed with an FC500 cytometer and CXP software (Beckman Coulter). Fluorescence gating parameters were established using antibody isotype controls, and values above the 99% negative staining threshold were considered positive. A total of 300.000 leukocytes were analyzed. Intermediate monocytes (CD14++CD16+) were identified by high-level expression of CD14 and were further distinguished from classical monocytes (CD14++CD16-) by co-expression of CD16. TEMs were identified within the intermediate subset by concomitant TIE2 expression (CD14++CD16+TIE2+ cells). Gating strategies are documented in Figure S1. Data are given in % frequency of blood leukocytes and mean fluorescence intensity (MFI). Conversion to absolute cell counts per ml blood was based on leukocyte concentrations established with an automated blood cell counter (Sysmex Corp., Kobe, Japan).

### Analysis of Soluble Blood Parameters

For plasma preparation, blood was drawn into pre-chilled tubes containing citrate, theophylline, adenosine and dipyridamole, and was processed on ice within 30 minutes as previously described [26]. Serum samples were kept at room temperature for 2 h to allow for efficient blood clotting, followed by centrifugation. Plasma and serum samples were stored in aliquots at –80°C for subsequent analysis. For measurement of ANG-2, sTIE1, MCP-1 (R&D Sytems, Inc., Minneapolis, MN, USA) and VEGF-A (Invitrogen Corp., Camarillo, CA, USA) commercially available ELISA kits were applied according to manufacturer’s instructions. The samples were assayed in duplicates with a Varioskan microplate reader (Thermo Fisher Scientific Inc., Waltham, MA, USA).

### Tumor Cell Culture

Tumor cell lines SW480, SW620 and HT-29 were obtained from the American Type Culture Collection (LGC Standards, Bury, UK). Cells were seeded at a density of 4x10^5^ per cm^2^ in growth medium with 10% fetal calf serum (FCS). After 24 hours, medium was changed to endothelial basal medium 2 (Lonza, Walkersville, MD) with 0.1% bovine serum albumin (BSA), and tumor cell supernatants were collected after another 24 hours in culture. Supernatants were centrifuged to remove cell debris and stored in aliquots at –20°C.

### *In vitro* Stimulation of PBMCs

Blood from healthy volunteers was drawn into EDTA tubes and peripheral blood mononuclear cells (PBMCs) were isolated with Ficoll Paque (GE Healthcare, Uppsala, Sweden). PBMCs were washed extensively to allow for a minimum of platelet contamination. 2x10^6^ PBMCs were seeded into each well of a 12-well plate (Corning Inc., Corning, NY, USA) and cells were exposed to tumor culture supernatant. Since serum-derived factors trigger the induction of CD16 expression on monocytes in culture, the addition of FCS was kept to a minimum of 1% supporting monocyte survival. After 24 hours of incubation, the non-adhering cell fraction was removed and adhering cells were harvested by scraping. Cells were stained for CD14 and CD16 and fixed in formaldehyde for flow cytometric analysis.

### Statistical Analysis

Statistical calculations were based on non-parametric tests using SPSS software version 17 (SPSS Inc., Chicago, IL, USA). Differences between the three study groups were assessed by applying the Wilcoxon-Mann-Whitney-U Test. Correlations between the investigated parameters were determined by Spearmańs rank correlation coefficient. Cancer prediction by independent variables was assessed in univariate and multivariate analysis with binary logistic regression. A receiver operating characteristic (ROC) curve served to define the cut-off value for CRC diagnosis. The reported p-values are results of two-sided tests. P-values ≤0.5 are considered statistically significant. Boxplots are depicted without extreme values to improve resolution.

## Results

### Intermediate Monocytes are Significantly Increased in CRC Patients

We applied flow cytometry to investigate the frequency of monocyte subpopulations in healthy individuals and colorectal cancer patients. The three collectives of healthy subjects (N = 32), patients with localized disease of stages I to III (N = 24) and patients with metastatic disease in stage IV (N = 37) showed comparable sex distribution but differences in their age range (Table 1).

**Table 1:** Demographic and clinical characteristics of study participants.

	Healthy	Localized CRC	mCRC
**Number, N**	32	24	37
**Sex, N (%)**			
Male	19 (59%)	12 (50%)	21 (57%)
Female	13 (41%)	12 (50%)	16 (43%)
**Age, years**			
Median	56	75	65
Range	42–74	48–88	42–80
**UICC N (%)**			
I	N.A.	6 (25%)	
II		6 (25%)	
III		11 (46%)	
IV		0 (0%)	37 (100%)
N.D.		1 (4%) *	

N: number of individuals; N.A.: not applicable; N.D.: not determined; UICC: Union for International Cancer Control staging.

* Patient with localized CRC was diagnosed based on biopsy material and computed tomography scan. Stage of disease was not determined, as patient did not undergo surgery.

**Figure 1:**
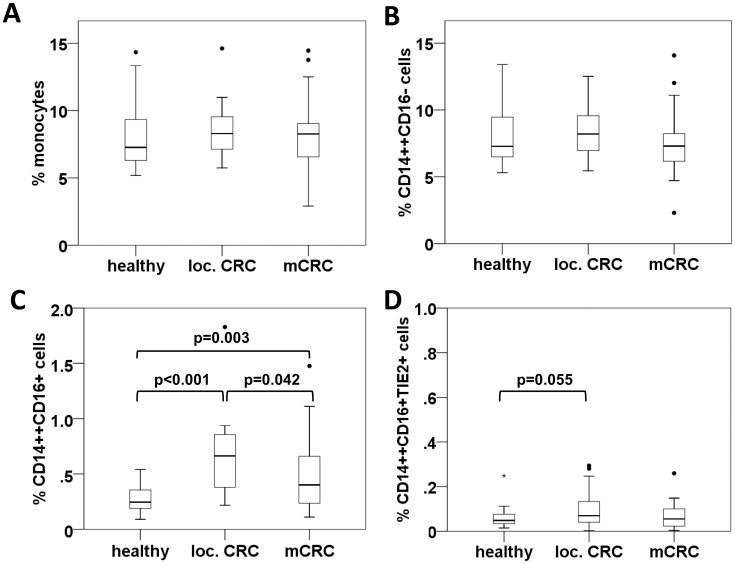
Intermediate monocytes are significantly elevated in CRC patients. Monocyte subpopulations were determined by flow cytometry and are given in % of total leukocyte counts in healthy individuals, patients with localized CRC and patients with mCRC. The frequency of total (CD14 positive) monocytes (A) was compared to classical CD14++CD16- monocytes (B), intermediate CD14++CD16+ monocytes (C), and CD14++CD16+TIE2+ TEMs (D).

The predominant population of classical (CD14++CD16−) monocytes and the total (CD14 positive) monocyte count did not differ between groups (Fig. 1). In contrast, intermediate (CD14++CD16+) monocytes were 2.6-fold increased in patients with localized CRC compared to healthy volunteers (p<0.001) ranging at a median level of 0.66% versus 0.25% of total blood leukocytes, respectively. In advanced disease, intermediate monocytes decreased again resulting in a median frequency of 0.40% in metastatic CRC patients, but levels remained significantly higher than in healthy controls (p = 0.003). When evaluating intermediate monocytes in cell counts per ml peripheral blood or in percentage of total monocytes, comparable results were obtained (data not shown).

Similarly, the (CD14++CD16+TIE2+) TEM subset of monocytes showed a non-significant tendency to rise in local disease (p = 0.055) with a median frequency of 0.05%, 0.07%, and 0.05% in total blood leukocytes for healthy, local CRC and mCRC collectives, respectively. Evaluation of TEMs based on the co-expression of CD14+TIE2+ as opposed to triple gating for CD14++CD16+TIE2+ did not alter results (data not shown).

### Blood Levels of Intermediate Monocytes are a Diagnostic Indicator of CRC

**Figure 2:**
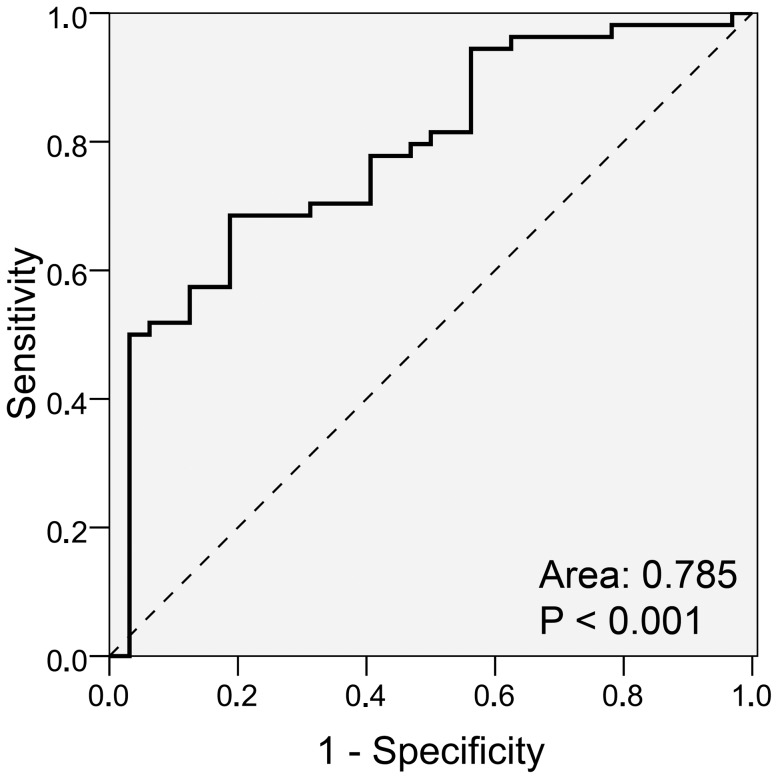
Blood levels of intermediate monocytes are a diagnostic indicator of CRC. The balance in diagnostic sensitivity and specificity of intermediate monocyte levels was evaluated by ROC chart and the area under the curve.

We further evaluated the performance of intermediate monocytes in CRC diagnosis irrespective of disease stage. ROC analysis was applied to determine the diagnostic power by the area under the curve (AUC = 0.785, p<0.001) and to establish a threshold for cancer detection (Fig. 2). With a cut-off value at 0.37% intermediate monocytes of total leukocytes the diagnostic sensitivity and specificity ranged at 69% and 81%, respectively.

### Monocytes of CRC Patients Show Altered Expression Levels of CD14 and CD16

The mean fluorescence intensity (MFI) of flow cytometric measurements reflects the average expression level of the investigated surface molecules on cells and is linked to monocyte function. While CD14 expression correlates with monocyte responsiveness and cytokine secretion [27], their CD16 levels are associated with their capacity for antibody-dependent cellular cytotoxicity [28].

**Figure 3:**
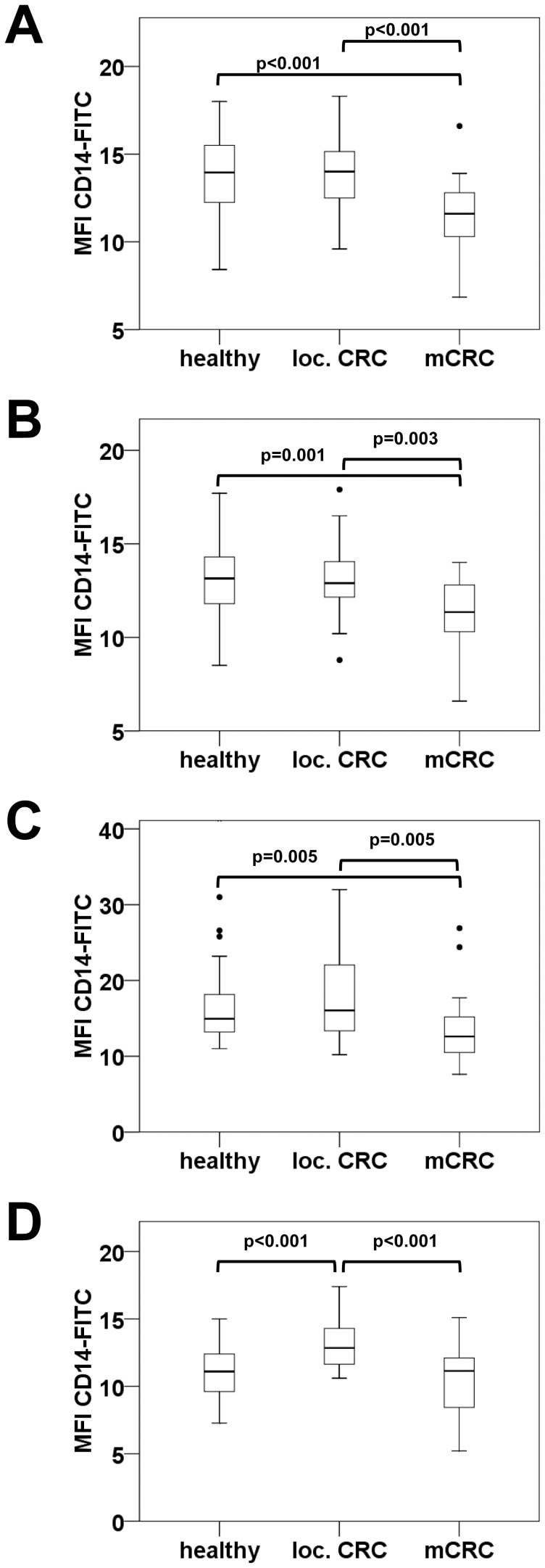
CD14 expression of all monocyte populations decreases in the advanced stage of mCRC. CD14 expression levels in healthy individuals, in patients with localized CRC or mCRC were assessed by mean fluorescence intensity (MFI) of CD14-FITC staining on total (CD14 positive) monocytes (A) or on the subsets of classical CD14++CD16− monocytes (B), intermediate CD14++CD16+ monocytes (C), and CD14++CD16+TIE2+ TEMs (D).

**Figure 4:**
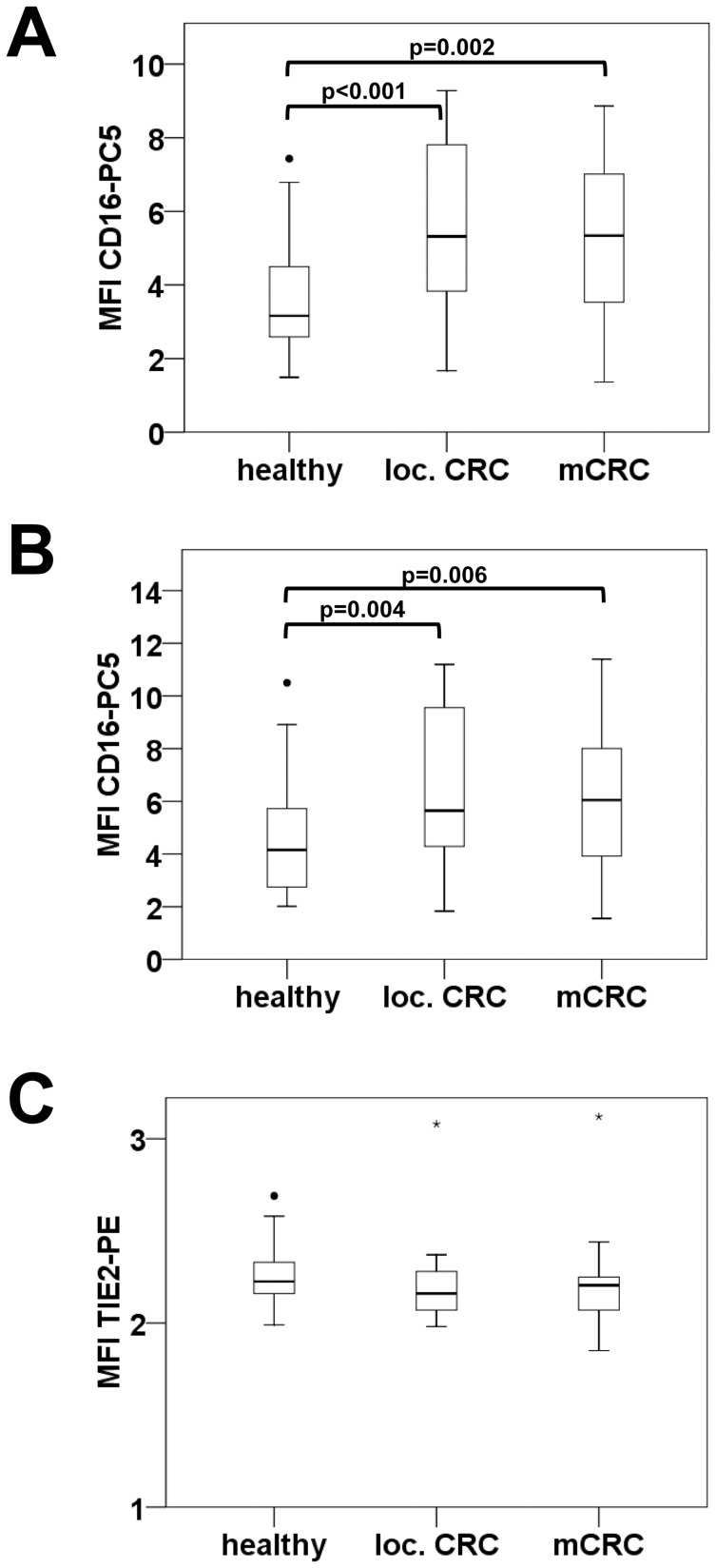
CD16 expression is increased on monocytes of cancer patients. The expression level of CD16 and TIE2 on monocyte subsets was determined for healthy individuals and patients with localized or metastatic colorectal cancer. Mean fluorescence intensity (MFI) of CD16-PC5 staining was evaluated for intermediate CD14++CD16+ monocytes (A), and CD14++CD16+TIE2+ TEMs (B). TIE2 expression levels are reflected by MFI of TIE2-PE antibody bound to TEMs (C).

We found that total monocytes showed a significant loss of CD14 in the advanced stage of metastatic CRC (Fig. 3). This CD14 reduction was also observed when separately evaluated for classical and intermediate monocytes. Within the subpopulation of TEMs highest CD14 expression levels were recorded in local disease. Intermediate monocytes and TEMs additionally revealed significantly enhanced expression levels of CD16 in cancer patients compared to healthy volunteers (Fig. 4), whereas TIE2 expression levels of TEMs were not altered in disease.

### Intermediate Monocytes are an Independent Predictor of Colorectal Cancer

**Figure 5:**
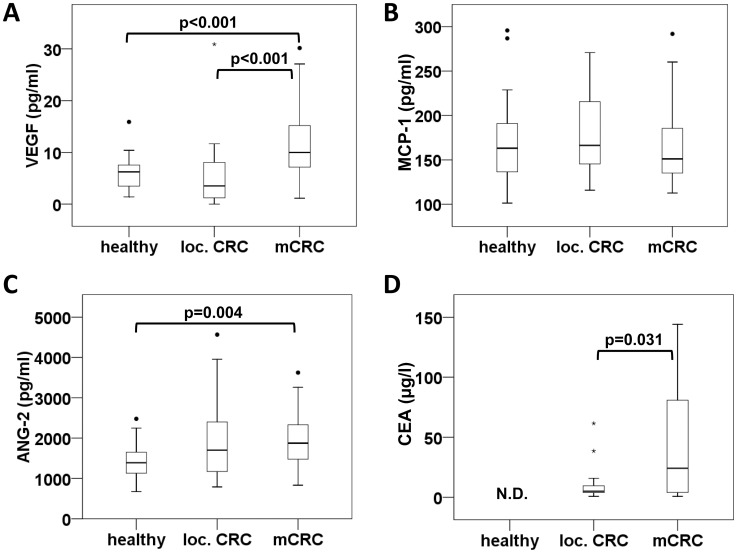
Angiogenesis factors are significantly elevated in mCRC while MCP-1 blood levels are not increased. Soluble parameters including VEGF-A (A), MCP-1 (B) and ANG-2 (C) were measured in plasma samples of healthy individuals, patients with localized CRC or mCRC. Blood concentrations of the tumor marker CEA (D) were available from routine hospital analysis of cancer patients but were not determined (N.D.) for the healthy control collective.

To evaluate whether cancer-related changes in monocyte subpopulations may be associated with factors known to regulate the recruitment and activation of intermediate monocytes and TEMs, we investigated blood levels of MCP-1, ANG-2, sTIE1 and VEGF-A in healthy volunteers and CRC patients. The analysis of soluble parameters in plasma showed a strong increase in VEGF-A (p<0.001) and ANG-2 (p = 0.004) in patients with metastasized disease (Fig. 5) whereas soluble TIE1 levels did not significantly differ between groups (data not shown). MCP-1 concentrations were determined in plasma (Fig. 5) and in a subset of serum samples (data not shown) but did not yield any significant differences between the three investigated collectives. For comparison, the tumor marker CEA (as established by routine hospital analysis) was evaluated in patients and revealed a significant increase of CEA blood levels in mCRC as compared to localized cancer thus documenting the advanced stage of disease. Of note, none of the investigated plasma parameters showed a significant correlation with monocyte subpopulations.

Logistic regression analysis was further performed to assess the significance of the available parameters in cancer prediction. Table 2 specifies the respective significance levels and odds ratios with 95% confidence intervals. Univariate analysis revealed age, plasma, VEGF-A and the blood levels of CD14++CD16+ intermediate monocytes (p = 0.004, odds ratio = 22) as predictors for colorectal cancer. In multivariate analysis, patient age and intermediate monocytes (p = 0.024, odds ratio = 83) prevailed as significant independent variables in cancer prediction.

**Table 2:** Univariate and multivariate analysis by binary logistic regression to calculate significance levels and odds ratios (including 95% confidence intervals) for cancer prediction by the investigated variables.

Parameter	Univariate Analysis			Multivariate Analysis		
	Significance	Odds Ratio	95% CI	Significance	Odds Ratio	95% CI
**Age**	**<0.001**	**1.11**	**1.05–1.17**	**0.003**	**1.15**	**1.05–1.27**
Sex	0.627	0.81	0.34–1.92	0.330	0.51	0.13–1.97
**VEGF**	**0.049**	**1.09**	**1.00–1.20**	**0.073**	**1.14**	**0.99–1.31**
sTIE1	0.240	1.04	0.97–1.12	0.555	0.97	0.86–1.09
ANG-2	0.145	1.00	1.00–1.00	0.818	1.00	1.00–1.00
MCP-1	0.566	1.00	1.00–1.00	0.147	0.99	0.98–1.00
% CD14+	0.420	1.07	0.90–1.28	0.114	0.47	0.19–1.20
% CD14++ CD16−	0.806	1.02	0.86–1.22	0.343	1.56	0.62–3.91
**% CD14++ CD16+**	**0.004**	**22.2**	**2.64–186**	**0.024**	**83.0**	**1.79–3842**
% CD14++ CD16+ Tie2+	0.132	779	0.13–4502897	0.331	0.03	0.00–31.1

### CD16 Expression on Monocytes is Induced by Colon Cancer Cells *in vitro*

**Figure 6:**
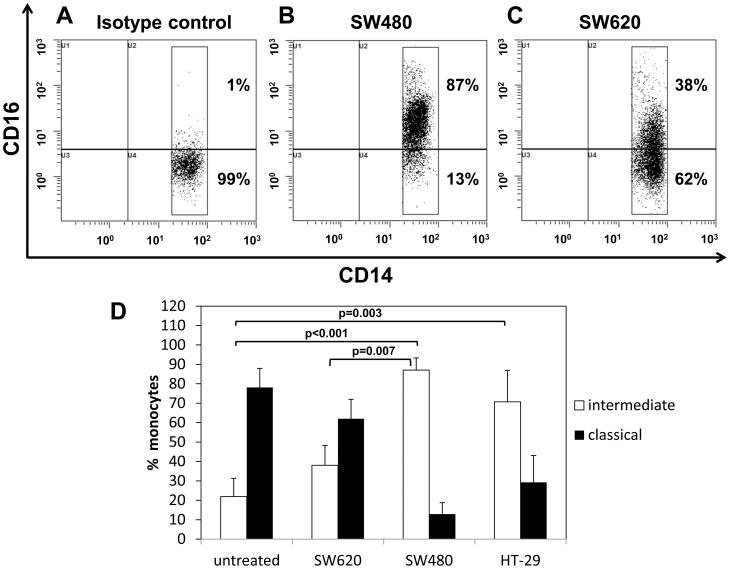
CD16 on CD14++ blood monocytes is induced *in vitro* by tumor-derived stimuli. PBMCs were isolated from healthy volunteers and incubated for 24 hours with supernatant of tumor cell cultures derived from CRC lines HT-29, SW480 and SW620. Adherent cells were harvested and stained with CD14-FITC and CD16-PC5 antibodies; mIgG_1_-PC5 was used for isotype control. Flow cytometric data of one representative experiment are illustrated for isotype control (A) versus CD16-PC5 staining of SW480 (B), and SW620 (C) stimulated PBMC cultures. Mean and standard deviation of three independent experiments with different blood donors are given in a bar diagram (D) illustrating the percentage of CD16+ (intermediate) versus CD16- (classical) cells within the CD14++ monocyte population.

Since none of the investigated plasma parameters correlated with the induction of intermediate monocytes in cancer patients, we aimed to examine by *in vitro* experiments whether tumor-derived stimuli can indeed trigger CD16 expression on blood monocytes and increase the frequency of the intermediate subset. PBMCs were isolated from healthy volunteers and analyzed for CD14 and CD16 expression after 24 h of incubation with tumor cell supernatants. We compared stimulation with supernatants derived from primary (SW480 and HT-29) and metastatic (SW620) colon cancer lines. At baseline, intermediate monocytes constituted 22% of CD14++ cells (Fig. 6). After 24 hours of *in vitro* incubation with supernatant from primary-derived (SW480 and HT-29) CRC cells, the frequency of intermediate CD14++CD16+ monocytes was significantly increased to 71–87%. Interestingly, PBMC incubation with the supernatant of the metastatic colon cancer line SW620 resulted in a moderate rise to 37% intermediate monocytes among CD14++ cells after 24 h.

## Discussion

Monitoring of monocyte subpopulations in peripheral blood has originally been based on the simple detection of CD14 and CD16 surface markers [10], [23], [29]. This has led to the conclusion that CD16+ monocytes constitute about 10% of total blood monocytes and amount to 50 cells per microliter of whole blood [30]. A two-fold increase in this inflammatory monocyte subset is observed during systemic infections, correlates with disease severity and is restored upon patient treatment and recovery [31]–[33].

An advanced protocol for the evaluation of monocyte subsets was released in 2010 [34] based on the concomitant detection of HLA-DR, CD14, and CD16 to avoid interference by granulocyte and natural killer cell populations. In our study, we chose to focus on the intermediate CD14++CD16+ and the TIE2-expressing monocyte populations with the marker combination of CD14, CD16, and TIE2. It should be noted that this detection approach does not allow for the unequivocal detection of non-classical monocytes which may be “contaminated” by natural killer cells and which have therefore been omitted from this analysis. A comparable staining strategy has previously been reported for successful TEM detection within the intermediate monocyte subset [35]. We assessed the concentration of these monocyte populations in the blood of healthy volunteers and detected a median level of 16 (interquartile range 11–30) intermediate CD14++CD16+ monocytes and 3 (interquartile range 2–6) TEMs per microliter of peripheral blood. When calculated in percentage, the intermediate and TEM subsets amounted to 0.25% and 0.05% of total blood leukocytes and to 3.39% and 0.63% of monocytes, respectively.

The aim of our study was to determine whether these monocyte subpopulations are elevated in colorectal carcinoma patients and have a diagnostic marker potential. We found the subset of intermediate monocytes to be 2.2-fold elevated in disease to a median level of 0.55% of blood leukocytes in CRC patients (0.66% in localized disease and 0.40% in metastatic disease). ROC analysis led to the selection of a cut-off point at 0.37% which resulted in a diagnostic sensitivity of 69% and specificity of 81%. The predictive value of this monocyte subset was further confirmed by multivariate regression analysis including all available parameters. Apart from patient age, intermediate monocytes were the only significant independent variable for cancer prediction. In this context it should be noted, that the recruited collectives of healthy individuals and cancer patients differed in median age (56 and 68 years, respectively) – a bias which is accounted for in multivariate analysis.

The limitation of monocyte populations in cancer diagnosis is given by the fact that fluctuations of monocyte counts in blood are also observed for other conditions, in particular inflammatory diseases [20], [22]. The blood profile of monocyte subsets might therefore reflect an epiphenomenon unrelated to the tumor. To address this issue, we have compared leukocyte counts among our study groups, since an increase in leukocyte concentration is commonly associated with infectious and inflammatory conditions. There was no significant difference between the healthy individuals and colorectal cancer patients investigated in our study (Fig. S2). Furthermore, blood levels of the inflammatory marker C-reactive protein (CRP) were available from routine hospital analysis of cancer patients. While CRP levels correlated significantly with leukocyte counts (P<0.001; k = 0.474), there was no correlation between CRP levels or leukocyte concentrations with intermediate monocyte counts. These findings support the notion that the increased level of intermediate monocytes in cancer patients was not induced by undiscovered inflammatory conditions in our study.

Previously published analyses of cancer patients were focused on the simple discrimination between CD16 positive or negative monocytes, i.e., were combining the non-classical and intermediate cell populations in their detection strategy. The CD16+ monocyte subset was reported to be significantly elevated in patients with malignant disease [36]. In particular, a recent study on breast cancer showed that CD16+ monocytes were 1.7-fold increased in patients as compared to healthy controls. The authors noted that levels were higher in stage I than stage II–IV disease [10]. Comparably, we found the intermediate CD14++CD16+ monocyte subset to be significantly elevated in colorectal cancer patients, but to show higher numbers in local than metastatic disease. In addition to the frequency of intermediate monocytes, their mean expression of CD16 increased, potentially enhancing their capacity for antibody-dependent cellular cytotoxicity [28].

The expression of CD16 on monocytes can be induced by cytokines such as MCP-1 [10], TGF-β [37], or M-CSF [11]. Conversely, this effect can be blocked by Th2-type cytokines like IL-4 [12] and by GM-CSF [37]. Thus, it is conceivable that the increase in intermediate (CD14++CD16+) monocytes is more actively triggered by the primary tumor, while tumor progression in metastatic disease results in a Th2-type cytokine milieu with counteracting effects. When screening our patient collectives for MCP-1 blood levels we did not detect any increase or correlation with the frequency of intermediate monocytes. However, other tumor-derived factors may account for the higher number of intermediate (CD14++CD16+) monocytes observed in cancer patients. In line, our *in vitro* analyses showed that incubation of PBMCs with tumor cell culture supernatants results in the rapid induction of CD14++CD16+ monocytes. Remarkably, this increase was less pronounced in response to culture supernatant from the metastatic cell line SW620 derived from the same patient as the primary carcinoma cell line SW480 or the unrelated primary CRC line HT-29. While these *in vitro* experiments do not identify the origin of CD14++CD16+ monocyte induction *in vivo*, they support the notion that intermediate monocytes may be more effectively induced in early stages of disease.

CD16+ monocytes are reportedly the main monocyte population in the anti-cancer response and secrete high levels of TNF-α and IL-12 upon tumor cell interaction [38]. However, the functional distinction between non-classical and intermediate monocytes is missing in this context. The fact that intermediate monocytes may express pro-angiogenic (and thus potentially pro-tumor) factors such as TIE2, endoglin or VEGFR-2 has prompted us to investigate a particular subset of intermediate monocytes, the CD14++CD16+TIE2+ TEMs. While potent tumor-promoting effects have been demonstrated for TEMs in mouse experiments [16], [39], clinical studies on their marker potential in cancer patients are largely missing [17]. In line with a very recent report on TEM frequencies in colorectal carcinoma [40], we found that TEMs were not significantly elevated in the blood of colorectal cancer patients and showed no correlation with the circulating angiogenesis factors ANG-2, sTIE1 and VEGF-A known to regulate TEM recruitment or activity [18]. Although we did not detect a significant difference in TEM counts between healthy individuals and cancer patients, we observed a trend for higher TEM levels in localized disease. The conclusion that circulating TEMs constitute a poor diagnostic marker for colorectal cancer may therefore be due to a smaller increase in TEM levels as compared to intermediate monocyte counts in disease. A larger collective of study participants would be required to determine whether this difference is of statistical significance.

In summary, when comparing intermediate CD14++CD16+ monocytes and CD14++CD16+TIE2+ TEMs in the blood of 32 healthy subjects and 61 CRC patients, we found that intermediate monocytes as opposed to TEMs were significantly elevated in disease. The highest frequency of CD14++CD16+ cell counts was, intriguingly, recorded for localized as opposed to metastatic disease which may reflect the preferential induction of intermediate monocytes in the early stages of cancer – a concept which was supported by *in vitro* studies. The frequency of intermediate monocytes was found to be an independent predictor of colorectal cancer. While taking into consideration that intermediate monocytes may also be elevated in other inflammatory conditions, this parameter may prove a valuable addition to the established CRC diagnosis markers by reflecting the early immune response against the tumor. It represents a less invasive screening option which should further be evaluated in a larger patient collective in support of colonoscopy and other diagnostic tests, in particular to detect the early stages of disease.

## Supporting Information

Figure S1 **Gating strategy for the detection of monocyte subpopulations by flow cytometry.** (A) Leukocytes (gate P1) were detected in a forward (FS) and side scatter (SS) diagram showing lymphocyte (L), monocyte (M) and granulocyte (G) populations. (B) CD14-FITC and CD16-PC5 positive cells were then identified among leukocytes. Classical monocytes (CD14++CD16-, gate P3) and intermediate monocytes (CD14++CD16+, gate P2) were clearly identified, whereas non-classical monocytes (CD14+CD16++, dashed gate) partially overlapped with the CD14-CD16++ natural killer (NK) cell subset and were not included in the analysis. (C) TIE2 expression (black line) was measured with reference to the intermediate monocyte subset (P2 gating) to detect TEMs (CD14++CD16+TIE2+, gate P4) and was evaluated in a histogram in comparison to immunolabeling with mouse IgG_1_-PE isotype control (grey line). A total of 300.000 leukocytes (P1) were analyzed, and the counts of monocyte subsets (P2, P3, P4) were expressed in % of all leukocytes.
(TIF)

Figure S2 **Leukocyte concentrations are comparable between the study collectives of healthy individuals and cancer patients.** Leukocyte concentrations were determined in the peripheral blood of healthy individuals and colorectal cancer patients with localized or metastatic disease.
(TIF)
