# Supplementary figures and images for: Intermediate Monocytes but Not TIE2-Expressing Monocytes Are a Sensitive Diagnostic Indicator for Colorectal Cancer
Source: PLoS One. 2012 Sep 4;7(9):e44450. doi: 10.1371/journal.pone.0044450 (PMC3433422; doi:10.1371/journal.pone.0044450)

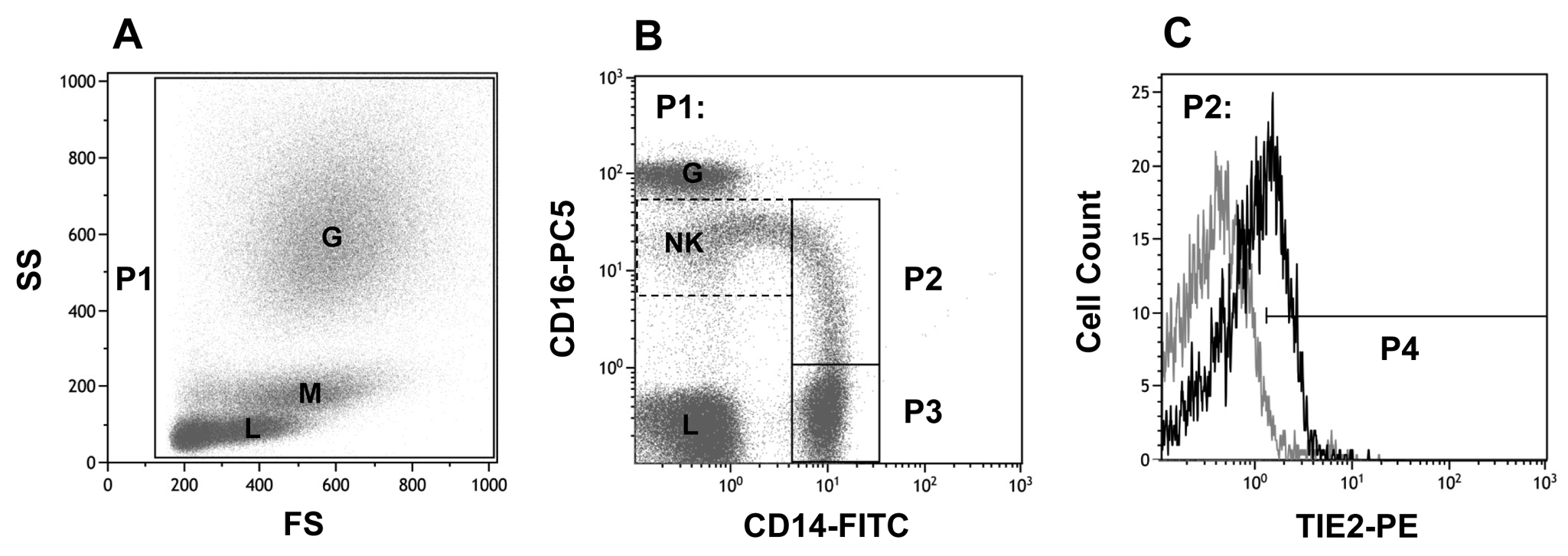

Supplement: Figure S1 — Gating strategy for the detection of monocyte subpopulations by flow cytometry. (A) Leukocytes (gate P1) were detected in a forward (FS) and side scatter (SS) diagram showing lymphocyte (L), monocyte (M) and granulocyte (G) populations. (B) CD14-FITC and CD16-PC5 positive cells were then identified among leukocytes. Classical monocytes (CD14++CD16-, gate P3) and intermediate monocytes (CD14++CD16+, gate P2) were clearly identified, whereas non-classical monocytes (CD14+CD16++, dashed gate) partially overlapped with the CD14-CD16++ natural killer (NK) cell subset and were not included in the analysis. (C) TIE2 expression (black line) was measured with reference to the intermediate monocyte subset (P2 gating) to detect TEMs (CD14++CD16+TIE2+, gate P4) and was evaluated in a histogram in comparison to immunolabeling with mouse IgG1-PE isotype control (grey line). A total of 300.000 leukocytes (P1) were analyzed, and the counts of monocyte subsets (P2, P3, P4) were expressed in % of all leukocytes. (TIF) [file pone.0044450.s001.tif]

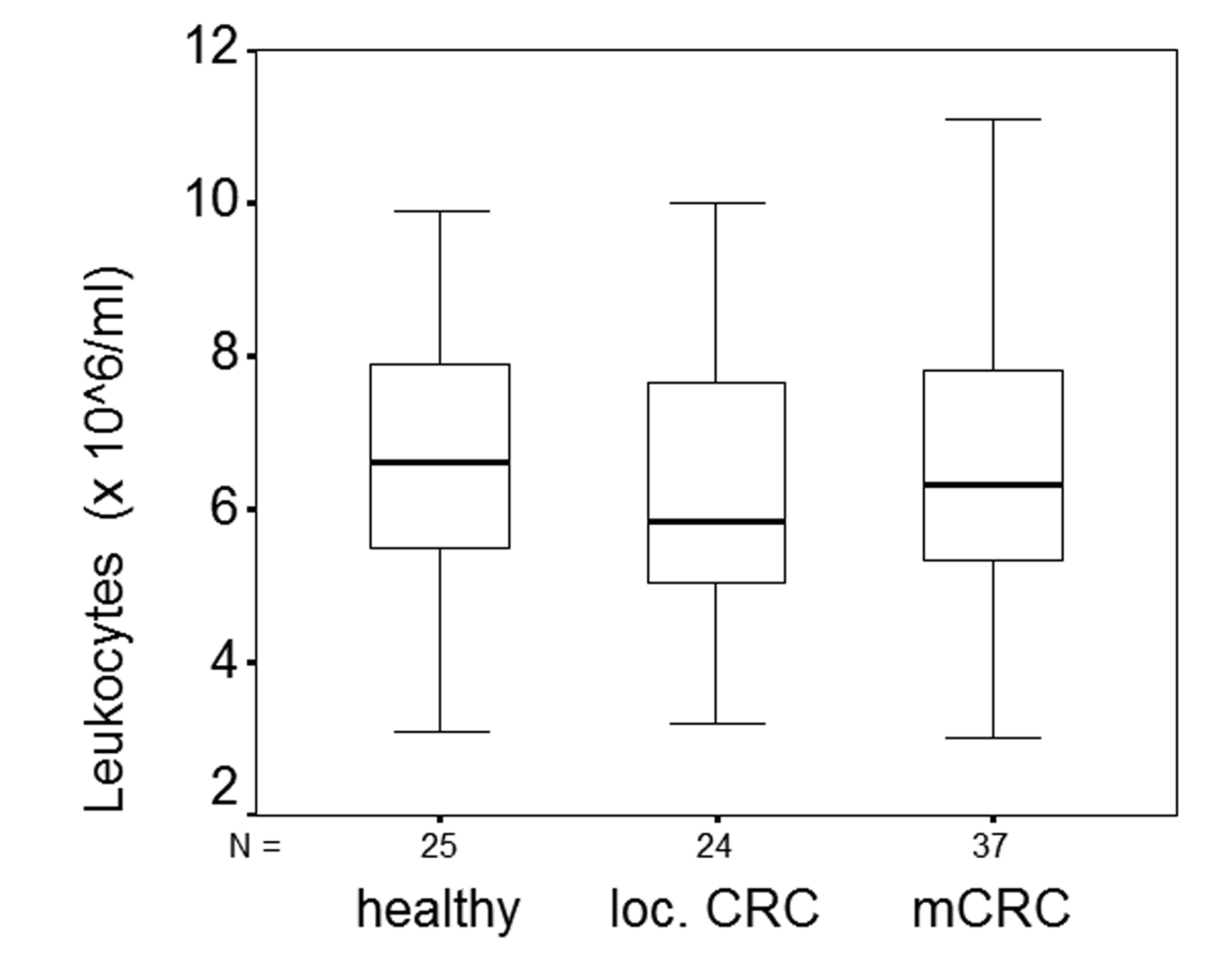

Supplement: Figure S2 — Leukocyte concentrations are comparable between the study collectives of healthy individuals and cancer patients. Leukocyte concentrations were determined in the peripheral blood of healthy individuals and colorectal cancer patients with localized or metastatic disease. (TIF) [file pone.0044450.s002.tif]
